# A Randomized Controlled Trial Comparing the Attention Training Technique and Mindful Self-Compassion for Students With Symptoms of Depression and Anxiety

**DOI:** 10.3389/fpsyg.2018.00827

**Published:** 2018-05-25

**Authors:** Ragni B. Haukaas, Ingrid B. Gjerde, Grunde Varting, Håvard E. Hallan, Stian Solem

**Affiliations:** Department of Psychology, Norwegian University of Science and Technology, Trondheim, Norway

**Keywords:** attention flexibility, attention training technique, mindful self-compassion, depression, anxiety, mindfulness, metacognitive therapy, RCT

## Abstract

The Attention Training Technique (ATT) and Mindful Self-Compassion (MSC) are two promising psychological interventions. ATT is a 12-min auditory exercise designed to strengthen attentional control and promote external focus of attention, while MSC uses guided meditation and exercises designed to promote self-compassion. In this randomized controlled trial (RCT), a three-session intervention trial was conducted in which university students were randomly assigned to either an ATT-group (*n* = 40) or a MSC-group (*n* = 41). The students were not assessed with diagnostic interviews but had self-reported symptoms of depression, anxiety, or stress. Participants listened to audiotapes of ATT or MSC before discussing in groups how to apply these principles for their everyday struggles. Participants also listened to audiotapes of ATT and MSC as homework between sessions. Participants in both groups showed significant reductions in symptoms of anxiety and depression accompanied by significant increases in mindfulness, self-compassion, and attention flexibility post-intervention. These results were maintained at 6-month follow-up. Improvement in attention flexibility was the only significant unique predictor of treatment response. The study supports the use of both ATT and MSC for students with symptoms of depression and anxiety. Further, it suggests that symptom improvement is related to changes in attention flexibility across both theoretical frameworks. Future studies should focus on how to strengthen the ability for attention flexibility to optimize treatment for emotional disorder.

## Introduction

Anxiety and depression are the most common psychological disorders, with a lifetime prevalence of 28.8 and 16.6%, respectively (Kessler et al., 2005). Cognitive Behavioral Therapy (CBT; Beck, 1976) is often a recommended treatment for these disorders. However, meta-analyses indicate that the effect of common psychotherapies including CBT has probably been overestimated (Cuijpers et al., 2016). Thus, increased understanding for emotional disorder and further research on effective treatment is needed. The “third wave” CBTs, including Metacognitive Therapy (MCT; Wells, 2009) and mindfulness-based interventions, represent promising perspectives for understanding and treating these disorders. Attention Training Technique (ATT; Wells, 2009) is an auditory task developed as part of MCT, aiming at increasing attention flexibility. Mindful Self-Compassion (MSC; Germer and Neff, 2013; Neff and Germer, 2013) originates from the mindfulness tradition, with an explicit focus on relating to oneself in a friendly manner. Both ATT and MSC represent promising methods for group-administered intervention, with the benefit of being cost effective and easy to administer.

### Metacognitive therapy and attention training technique

MCT builds upon the self-regulatory executive function (S-REF) model, which seeks to explain cognitive and metacognitive factors involved in top-down control and maintenance of psychological disorders (Wells and Matthews, 1996). According to this model, cognitive processes are spread across three interconnected levels: low-level automatic and reflexive processing, cognitive style in the form of conscious processing of thoughts and behaviors, and metacognitive knowledge or beliefs stored in long-term memory. Metacognition refers to awareness and cognition about cognitive processes and includes cognitive factors that control, monitor, and appraise thinking (Wells, 2009).

According to the S-REF model, psychological disorder is linked to a perseverative style of thinking called the cognitive attentional syndrome (CAS; Wells, 2009). CAS consists of prolonged worry or rumination, threat monitoring, and different unhelpful coping styles accompanied by a heightened self-focused attention. This may lead to sustained dysfunctional processing, *reduced attentional flexibility*, and an experience of uncontrollability of negative thoughts and emotions. The aim in MCT is to eliminate the CAS and to modify dysfunctional metacognitive beliefs about control, appraisal, and cognitive and emotional processing, and thereby strengthen the ability to react in a more flexible way to negative internal stimuli. ATT is one method for achieving this.

A meta-analysis of MCT for anxiety and depression including 16 studies demonstrated large effect sizes and suggested that MCT might be superior to CBT (Normann et al., 2014). Later randomized controlled trials (RCTs) also support the effectiveness of MCT in treating depression (e.g., Jordan et al., 2014; Hagen et al., 2017) and anxiety disorders (e.g., Johnson et al., 2017), and preliminary results indicate that MCT may be suited for group administration (Dammen et al., 2015; Papageorgiou and Wells, 2015).

ATT is a component of MCT designed to strengthen attentional control and promote external focus of attention, to interrupt and break free of the CAS (Wells, 2009). The exercise is auditory, and consists of three sections targeting different attentional components: selective attention, attention switching, and divided attention. The aim is not to distract oneself from difficult thoughts or feelings, but rather to increase flexibility and thus voluntarily being able to choose attentional focus.

Although originally a part of MCT, a growing number of studies are examining the potential of ATT as a standalone intervention. A systematic review with meta-analytic elements summarizes findings from 10 ATT-studies including four studies with a single case experimental design, four RCTs, and two case studies (Knowles et al., 2016). Number of ATT-sessions ranged from 1 to 11 sessions among the included studies. Although still preliminary, the meta-analysis indicates that ATT may be effective in treating a wide range of psychological problems. Four of the included RCTs (Sharpe et al., 2010; Fergus et al., 2014; Nassif and Wells, 2014; Callinan et al., 2015) involved non-clinical samples and used one or two sessions, and are as such comparable to the current study. Within-group effect sizes pre- to post-intervention in these RCTs were medium to large: negative affect (*d* = 1.03), anxiety measures (range: *d* = 0.32–0.65), intrusive thoughts (range: *d* = 1.06–1.33), hypervigilance to pain (*d* = 0.95), self-focused attention (range: *d* = 0.55–1.78), and attention flexibility (*d* = 0.61). Effect sizes were also large for differences between groups for symptom measures in three of the RCTs. Further, the authors called for more evaluations of ATT against comparable interventions, such as mindfulness based interventions, including follow-up intervals (Knowles et al., 2016).

A general goal of ATT is to increase attention flexibility, often referred to as part of or similar to the concept of attentional control (Callinan et al., 2015). Attentional control can be described as the ability to direct and control attention voluntarily (Derryberry and Reed, 2002). A study suggested that poor attentional control limits the ability of emotion regulation, whereas high attentional control allows the individual to more flexibly disengage and orient attention away from threatening information (Derryberry and Reed, 2002). Hence, poor control may leave the individual vulnerable to emotional disorder. Attentional control is also negatively correlated to state anxiety (Spada et al., 2010) and a possible moderator of the relationship between activation of the CAS and symptoms of emotional disorder (Fergus et al., 2012). It has been demonstrated that ATT can strengthen attentional control measured by self-reported attention flexibility, with medium to large between-group effect sizes (range: η_*p*_^2^ = 0.12–0.15) (Nassif and Wells, 2014; Callinan et al., 2015). This was consistent with performance on a more objective laboratory-based task of attentional control (Callinan et al., 2015). It has thus been suggested that attentional flexibility/control might be a transdiagnostic protective factor and a putative change mechanism of ATT (Fergus and Bardeen, 2016).

### Mindfulness and self-compassion

Originating in Buddhist traditions, mindfulness can be defined as “the awareness that emerges through paying attention on purpose, in the present moment, and non-judgmentally to the unfolding of experience moment by moment” (Kabat-Zinn, 2003, p. 145). Thus, mindfulness can be regarded as consisting of two components: self-regulation of attention toward current experiences, and relating to these experiences in an open, curious, and accepting stance (Bishop et al., 2004). Attentional processes are important in mindfulness, such as focusing on inner experiences of breathing and emotional sensations. Research also indicates that mindfulness training has the potential to modify and strengthen attention following regular training, such as enhancing the ability to voluntarily shift focus of attention (Jha et al., 2007; Zylowska et al., 2008; Hölzel et al., 2011).

There is increasing support for the beneficial effects of mindfulness-based interventions and treatments. Different programs have been developed and evaluated, such as mindfulness-based cognitive therapy (MBCT; e.g., Segal et al., 2002) and mindfulness-based stress reduction (MBSR; e.g., Kabat-Zinn, 1982, 1990). A meta-analysis including 39 studies demonstrated that MBCT, MBSR, or similar interventions were effective in reducing symptoms of depression and anxiety in clinical and non-clinical samples, with medium to large effect sizes (Hofmann et al., 2010).

Based on research indicating that self-compassion might be one of several key mechanisms accounting for the positive effects following mindfulness-interventions, a MSC-program has been developed (Germer and Neff, 2013; Neff and Germer, 2013). According to Neff (2003b), self-compassion consists of three interrelated components: self-kindness, a sense of common humanity, and mindfulness. In these terms, being self-compassionate means relating to oneself in a friendly and patient manner, understanding that pain and suffering is experienced by all humans, and being mindfully aware of painful experiences without over-identifying with them. Mindfulness in the context of self-compassion is described as awareness in a balanced way to one's *negative* thoughts and emotions, and is thus slightly more specific than mindfulness in general (Neff, 2003b). The MSC-program is originally designed as an intervention of eight weekly group meetings, for both clinical and non-clinical populations, aiming at enhancing self-compassion through informal (during daily life) and formal (sitting meditation) exercises (Germer and Neff, 2013; Neff and Germer, 2013).

Research indicates that self-compassion is inversely related to psychopathology (Barnard and Curry, 2011), and a meta-analysis summarizing 20 cross-sectional studies found large effect sizes for the negative relationship between self-compassion and stress, anxiety, and depression (MacBeth and Gumley, 2012). Altogether, this indicates that self-compassion might increase resilience against stress and be an important buffer against psychopathology. One RCT evaluating the MSC-program with a non-clinical sample, found the program to be effective compared to a waitlist control (Neff and Germer, 2013). Between-group effect sizes were large for self-compassion and depression, small for stress, and medium for remaining measures such as anxiety and mindfulness. Another study of particular relevance for the current study found promising results with a briefer self-compassion intervention with a non-clinical sample of 52 students (Smeets et al., 2014). The RCT compared a self-compassion program of three weekly meetings to a time-management control group and demonstrated large effect size for self-compassion (*d* = 1.19), medium effect sizes for optimism (*d* = 0.66), self-efficacy (*d* = 0.52), and reduction in rumination (*d* = 0.70), and small effect size for worry (*d* = 0.19). This indicates that a three-session trial may be sufficient for a therapeutic effect, such as improving well-being and resilience (Smeets et al., 2014). However, this study did not include follow-up assessment.

### Comparisons of ATT and mindfulness-based interventions

As presented above, ATT and mindfulness originate from different traditions and as such have several dissimilarities. Meditation for instance, which is a core element of mindfulness-based interventions, is not recommended in MCT (Wells, 2009). Furthermore, although attention is emphasized in both mindfulness and ATT, they seem to differ in their perspectives on the preferential locus or direction of attentional focus. Self-focused attention can be defined as: “An awareness of self-referent, internally generated information that stands in contrast to an awareness of externally generated information derived through sensory receptors” (Ingram, 1990, p. 156). Heightened self-focused attention has traditionally been associated with psychopathology, and is considered a core component shared by several psychological disorders, such as anxiety and depression (Ingram, 1990). ATT targets inflexible and excessive self-focused attention, aiming to increase attention flexibility, and switch to a more *external* attentional focus (e.g., Wells, 2009). In the mindfulness tradition, however, increased *internal* attentional focus has been suggested as an important change mechanism for achieving the beneficial effects of mindfulness training (Baer, 2009). Distinct functions of different types of self-focused attention has been proposed (Trapnell and Campbell, 1999), and a ruminative, self-critical self-focus, as described in MCT, is probably different from the reflective, experiential self-focus associated with the mindfulness tradition (Baer, 2009). Studies have also supported this notion (Watkins and Teasdale, 2004).

Despite the conceptual differences, both intervention perspectives seem promising in reducing symptoms of anxiety and depression, and may operate through related mechanisms such as attentional processes. Therefore, the relationship between them are of interest to explore. This was done in a RCT with 76 students, comparing ATT to mindfulness-based progressive muscle relaxation (MB-PMR) in a single-session trial (Fergus et al., 2014). Symptoms of cognitive and somatic anxiety were significantly reduced after one session, with medium to large effect sizes in both the ATT-group (range: *d* = 0.32–0.65) and the MB-PMR-group (range: *d* = 0.59–1.04)^1^. Heightened self-focused attention was related to less anxiety after MB-PMR, whereas heightened externally focused attention was related to less anxiety after ATT. However, the study was a single-session trail and lacked follow-up assessment. Despite its limitations, this RCT indicates that both perspectives are effective in reducing symptoms of anxiety, and that the effect of self-focused attention might depend on whether it is performed in a mindfulness-based context or not (Fergus et al., 2014).

The effectiveness of these perspectives in reducing anxiety symptoms via common processes has also been supported in a recent RCT comparing ATT and MB-PMR to a thought wandering control (TWC) with 81 high trait anxious individuals (McEvoy et al., 2017). There was a significant reduction in state anxiety after a single session, with large effect sizes in both experimental groups (ATT: *d* = 0.83; MB-PMR: *d* = 0.85). Contrary to previous findings (Fergus et al., 2014), the notion that internal vs. external shifts in attention is associated with symptom reduction was not supported in this study. Furthermore, cognitive flexibility as measured by an emotional Stroop task was not associated with anxiety reductions. This was inconsistent with their hypothesis, previous studies (e.g., Nassif and Wells, 2014) and metacognitive theory (Wells, 2009). However, changes in present-focused attention and metacognitive beliefs were potent change mechanisms across ATT and MB-PMR. The authors concluded that the two techniques are more similar than different and may influence symptom reduction via common mechanisms (McEvoy et al., 2017). A related study exploring the theoretical basis of such interventions also found that mindfulness and metacognitions share important elements, although they are distinguishable constructs (Solem et al., 2015).

In summary, metacognitive and mindfulness-based traditions offer viable treatment options and may contribute to increased understanding of emotional disorder through different theoretical perspectives on self-regulation and attentional processes (e.g., Hofmann et al., 2010; Normann et al., 2014). Specifically, both ATT and MSC can be considered promising cost effective and easy-to-administer interventions for preventing and reducing symptoms of anxiety and depression (Neff and Germer, 2013; Knowles et al., 2016). Such interventions are originally built upon separate constructs, but may be somewhat overlapping (Solem et al., 2015; McEvoy et al., 2017). Thus, comparing these perspectives is of interest, as well as exploring how underlying mechanisms such as attentional control relates to symptom reduction (Fergus and Bardeen, 2016). As few controlled studies have evaluated these interventions, and only two studies have compared ATT and a mindfulness-based intervention (Fergus et al., 2014; McEvoy et al., 2017), more RCTs are needed.

The current RCT therefore sets out to compare the efficacy of ATT and MSC in a three-session trial over 3 weeks, thus expanding results from Smeets et al. (2014), Fergus et al. (2014), and McEvoy et al. (2017). However, the current RCT aims at overcoming limitations in these studies. Fergus et al. (2014) and McEvoy et al. (2017) did not include measures of depression or mindfulness and Smeets et al. (2014) did not include measures of anxiety and depression. Therefore, measures of both anxiety and depression will be included in addition to theoretical construct measures. Due to lacking information about long-term effects in the previous studies, follow-up assessment will also be included. The aim of the current RCT is to test the following hypotheses:

H1: Both interventions will lead to a significant reduction in symptoms of anxiety and depression. H2: Both interventions will give a significant increase in mindfulness, attention flexibility, and self-compassion and treatment-responders will experience more change than non-responders on these measures.

## Methods

### Participants

**Figure 1:**
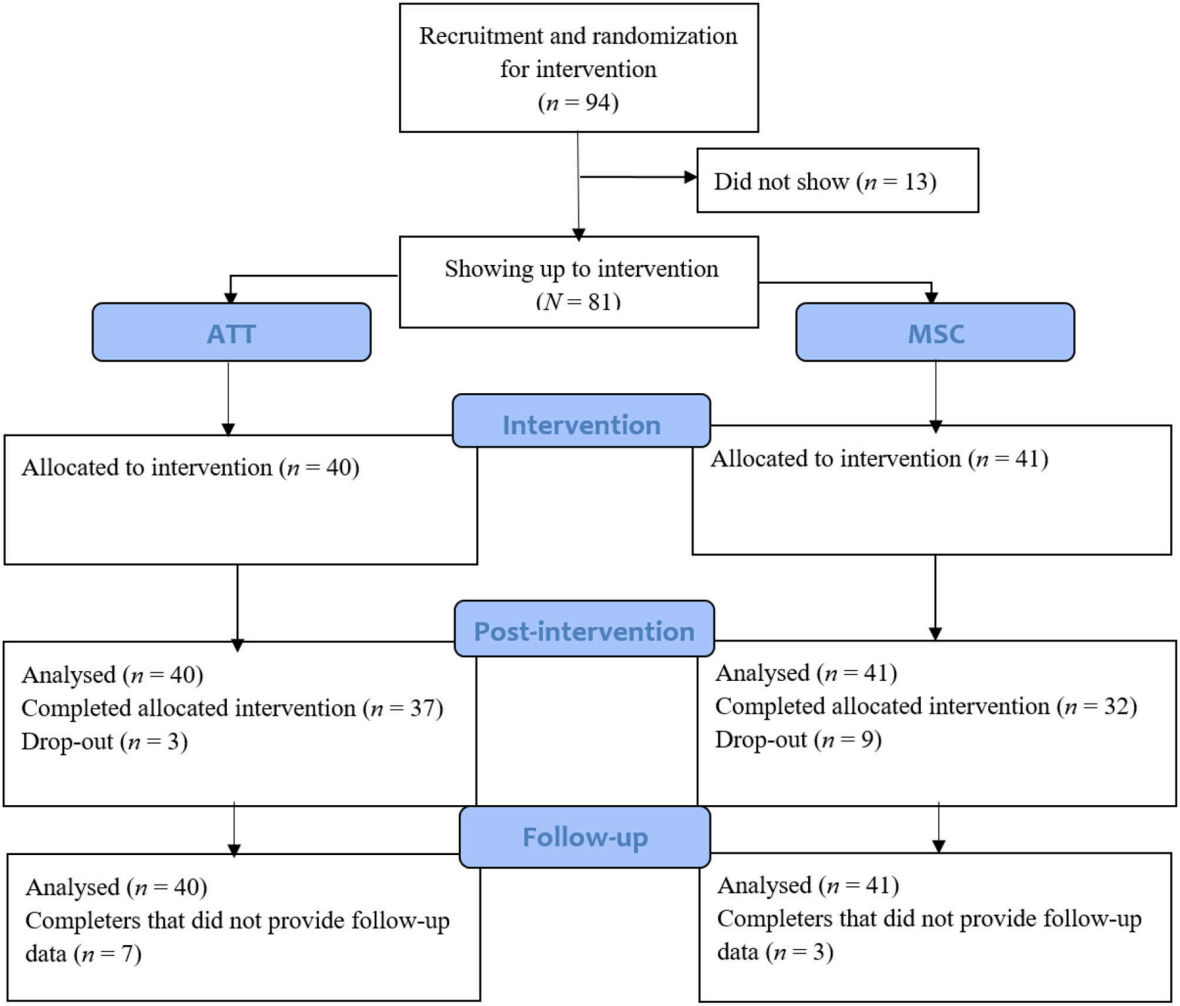
Flow chart presenting participant flow from assessment to follow-up.

A total of 94 participants showed interest in participation, of which 81 showed up to intervention. The total sample therefore consisted of 81 Norwegian undergraduate and graduate students at the Norwegian University of Science and Technology (NTNU), mean age 22.9 (*SD* = 3.3, range = 18–36). Participation was open for everyone interested. The participants were not assessed with diagnostic interviews, but had self-reported symptoms of depression, anxiety, and stress. The majority of the participants were female (75.3%) and 55.6% reported having a partner. In total, 69 participants completed the three-session intervention. A total of nine participants (22.0%) dropped out in the MSC condition and three participants (7.5%) in ATT, no reasons were reported. Participant flow is presented in Figure 1.

### Procedure

The study was a RCT approved by the Regional Medical Ethics Committee in Norway (ref.nr. 2015/470). Informed written consent was given from all participants. Intervention was implemented from 2015 to 2017. Participants were recruited at two NTNU campuses with flyers, posters, and promotion in lectures and social media. The study was presented as a course in stress management based on either mindfulness or attention training consisting of three group meetings. Participation was open for everyone interested, but the information implied that the course was suited for people experiencing excessive stress and worry. Using the *Research Randomize Program* (www.randomizer.org), the recruited participants were randomized to either the ATT or MSC experimental group. Participants were blind as to which experimental group they were allocated until the first group meeting. All participants completed an online questionnaire (described below) before, 1 week after, and at 6-month follow-up.

### Measures

The questionnaire consisted of demographics and three 1-item questions about level of test anxiety, self-esteem, and loneliness using a 4-point scale; general symptom measures of anxiety and depression used as primary outcome measures; and treatment-specific measures for evaluation of the constructs of mindfulness, self-compassion, and attention flexibility.

#### The patient health questionnaire-9 (PHQ-9; Kroenke et al., 2001)

PHQ-9 was used as a primary outcome measure in order to assess symptoms of depression. PHQ-9 is a 9-item self-report inventory based on DSM-criteria for depression. The items ask how often the individual has been bothered by symptoms (e.g., “Feeling down, depressed, or hopeless?”) over the last 2 weeks. Each item is rated on a 4-point scale (0 = *not at all*, 3 = *almost every day*). In the current study, PHQ-9 was used as a continuous measure with total scores ranging from 0 to 27, in which scores of 5, 10, and 15 represent mild, moderate, and severe depressive symptoms. Overall, the PHQ-9 has been shown to have good reliability and validity (Kroenke et al., 2001, 2010). In the current study, the PHQ-9 had a Cronbach's alpha of 0.83.

#### Generalized anxiety disorder-7 (GAD-7; Spitzer et al., 2006)

In order to evaluate level of anxiety symptoms, GAD-7 was used as a primary outcome measure. GAD-7 is a 7-item self-report questionnaire based on the DSM-criteria for generalized anxiety disorder. The items ask how often the individual has been bothered by symptoms (e.g., “Feeling nervous, anxious, or on edge?”) over the last 2 weeks. Each item is rated on a 4-point scale (0 = *not at all*, 3 = *almost every day*). GAD-7 total scores range from 0 to 21 wherein scores of 5, 10, and 15 may represent mild, moderate, and severe anxiety symptoms. Research has indicated good construct, criterion, factorial, and procedural validity, as well as good reliability, for GAD-7 (Spitzer et al., 2006). In the current study, the GAD-7 had a Cronbach's alpha of 0.82.

#### Self-compassion scale short form (SCS-SF; Raes et al., 2011)

The construct of self-compassion was measured using a short version of Neff's (2003a) original 26-item Self-Compassion Scale (SCS). The SCS-SF consists of 12 items being rated on a scale from 1 to 5 (1 = *almost never*, 5 = *almost always*). Items include e.g.: “I try to be understanding and patient toward those aspects of my personality I don't like,” and “When I feel inadequate in some way, I try to remind myself that feelings of inadequacy are shared by most people.” Total scores range from 12 to 60, in which higher score indicates higher self-compassion. As the original SCS, the SCS-SF measures six components of self-compassion: self-kindness, self-judgement, common humanity, isolation, mindfulness, and overidentification. The total scores in SCS-SF are almost perfect correlated with the original SCS, as well as having the same factor structure and good internal consistency (Raes et al., 2011). Good reliability has been found in non-clinical (Raes et al., 2011) and clinical (Lockard et al., 2014) samples. Raes et al. (2011) recommend the full SCS when subscale information is of interest. The current study uses total scores only. In the current study, the SCS-SF had a Cronbach's alpha of 0.86.

#### Detatched mindfulness questionnaire (DMQ; Nassif and Wells, 2007)

DMQ is a 22-item self-report measure assessing participants' different levels of awareness and how they respond to their thoughts. The measure consists of five theoretically derived constructs of detached mindfulness: attention flexibility, meta-awareness, detachment/observing self, thought control, and cognitive de-centering. These subscales are conceptualized as adaptive or maladaptive in the metacognitive model of psychological disorder. Each item is rated on a 5-point scale (1 = *disagree*, 5 = *agree*). In the current study, the subscale of particular interest was attention flexibility. This subscale has been used to measure the construct of attentional control/flexibility in previous ATT-studies (e.g., Nassif and Wells, 2014; Callinan et al., 2015). The subscale consists of five items, including e.g., “I am able to have a negative thought without worrying about it,” and “I can usually let go of my thoughts even if I'm worried.” Scores on this subscale can range from 5 to 25, with higher scores indicating higher levels of attention flexibility. In the current study, the DMQ flexibility subscale had a Cronbach's alpha of 0.80.

#### The five facet mindfulness questionnaire (FFMQ; Baer et al., 2006; Tran et al., 2013)

The FFMQ is a self-report measure assessing the following five facets of mindfulness: observing, describing, acting with awareness, non-judging of inner experience, and non-reactivity to inner experience. This five-factor solution was developed through factor analysis of combined items from five existing mindfulness questionnaires. Each item is rated on a 5-point Likert scale (1 = *never or rarely true*; 5 = *very often or always true*). An example is: “I perceive my feelings and emotions without having to react to them.” In the 39-item full form, the facets of FFMQ have demonstrated good reliability and validity (Baer et al., 2008; Christopher et al., 2012). The full FFMQ has also been validated for use in Norway (Dundas et al., 2013). In the current study, a 20-item short version (Tran et al., 2013) was used and FFMQ was reported as a total score. Total scores can range from 20 to 100, with higher scores indicating higher levels of mindfulness. In the current study, the 20-item FFMQ had a Cronbach's alpha of 0.80.

### Intervention

The intervention consisted of three group sessions 3 weeks in a row, of either ATT or MSC, as well as instructions to listen to certain audiotapes every day between sessions during the intervention period. Each group consisted of 6–10 participants sitting in a circle, being co-led by two therapists. All group sessions were held in the afternoon at the university campus and lasted for 45 min. The participants were instructed to take an active part in the group discussions while the therapists mainly were facilitating, asking socializing questions, and unraveling misunderstandings.

The following structure was used in the first two sessions in all groups:

1. Agenda-setting for the day.
2. Presenting the intervention condition: introducing the participants to the technique and their respective rationales.
3. Practicing the technique using pre-recorded audiotapes.
4. Discussion of the technique: in order to understand the exercise and how it can be useful and applied to everyday life.
5. Agreeing upon homework: listening to the audiotape between meetings. Forms were handed out so that participants could register practice frequency.

Session two and three in both conditions began by asking about homework, feedback from the participants, summing up the rationale, and unraveling possible misunderstandings. In the second session, this was followed by step three to four, as presented above.

In the third and last session, there was no practicing with audiotapes. Most of the time went to group discussion on the principles the participants had learned during the 3-week intervention. Each participant described in turn how they had experienced listening to the audiotapes and how they could relate the principles to their everyday life. Finally, participants provided evaluation feedback of the course. Differences between the conditions are described below.

#### ATT-intervention

After a brief discussion of self-focused attention and socialization based on a rationale for ATT (Wells, 2009, p. 59), the participants listened to a 12 min ATT audiotape together (available at: http://www.mct-institute.com/attention-training-technique). The exercise consists of six to nine sounds, in addition to a voice guiding the listener through three sections: 5 min of selective attention, 5 min of rapid attention switching, and 2 min of divided attention (Wells, 2009). The selective attention section consists of instructions to focus on individual sounds in an array of competing sounds at different spatial locations. This is followed by rapid attention switching between both spatial locations and the individual sounds with gradually increasing speed. The exercise concludes with a section of divided attention, in which the listener is instructed to expand his or her attention to process multiple sounds and locations simultaneously. The participants were instructed to focus on a visual fixation point during the exercise and not to use the audiotape as avoidance from uncomfortable thoughts and feelings.

A further discussion of the principles of ATT and experiences while listening to the audiotape followed. Using the Self-Attention Rating Scale (Wells, 2009, p. 267), participants were asked to rate their focus of attention before and after listening to the audiotape by choosing a number from −3 (indicating entirely externally focused) to +3 (entirely self-focused). Self-focused attention vs. external focus of attention was then discussed. Homework was ascribed in the form of listening to the audiotape once a day for 2 weeks. The participants could choose freely between listening to the original audiotape or a Norwegian translation. The last group session focused on use of the principles from ATT and general elements of metacognitive theory.

#### MSC-intervention

In the first session, the concept of mindfulness was introduced and general components such as moment-to-moment experiences, a non-judging attitude, and breathing were discussed. This was followed by listening to the first 10 min of a 20 min *Affectionate Breathing*-tape (available at: http://self-compassion.org/wp-content/uploads/2016/11/affectionatebreathing_cleaned.mp3), in which the listener is guided through a breathing exercise. The audiotape instructs the listener to keep an affectionate attitude and accept any arriving thought and urge. A brief discussion of the experience and principles in the exercise followed. As homework, participants were asked to listen to the full audiotape once a day and try to be mindful in their daily activities. The participants were instructed not to use the audiotape as avoidance or coping strategy.

In the second session, self-compassion was introduced for the first time after a short reminder of mindfulness. Following a brief discussion of self-compassion, the participants listened to the first 11.5 min of Neff's 20 min *Loving Kindness Meditation* (available at: http://self-compassion.org/wp-content/uploads/2016/11/LKM_cleaned.mp3). The aim is to generate compassion for oneself and others, and the audiotape instructs the listener e.g., to repeat compassionate phrases. After listening to the tape, experiences during the exercise were shared, and a further discussion followed on the differences between self-compassion and self-pity and how to relate to oneself in a kinder and more accepting manner. Homework was ascribed in the form of listening to the new full audiotape once a day until the next session and observing one's inner critical dialogue. The third and last session mainly focused on self-compassion and how to use the principles in everyday life.

### Therapists

The therapists were four clinical psychology students on their fifth year, under supervision from a clinical psychologist. The student therapists had no prior official training in ATT or MSC. Training involved extensive literature reading on the two conditions and receiving feedback on videotaped recordings of training sessions before the experimental group interventions started. Therapists also discussed and trained with the audiotapes themselves. All therapists conducted both ATT and MSC equally. The group leaders were supervised between the group sessions.

### Data analyses

To compare the two samples on demographics and measures pre-intervention, one-way analysis of variance (ANOVA) was used. Chi-square tests were used to compare the groups with respect to dichotomous variables.

Repeated measures ANOVA was used to assess changes in measures from pre- to post-intervention and follow-up, and a split-plot ANOVA was used to compare the groups with respect to changes in outcome measures. Pre-intervention, 2.5% values were missing on GAD-7, FFMQ, DMQ, and SCS-SF. Missing data in these measures were replaced by mean values. Last observation carried forward was used for missing data post-intervention for dropouts (7.5% for ATT and 22.0% for MSC) and missing follow-up data. To assess whether homework frequency influenced outcome, a split-plot ANOVA with number of homework exercises as a covariate was conducted. Missing data on the homework-variable was not replaced. Cohen's *d* with pooled *SD* and partial eta squared was calculated and reported as effect sizes. Cohen's *d* was calculated for each measure for each group, and correlations between pre- and post-intervention values were included in the calculation. This was done using Morris and DeShon's (2002) Equation (8), which corrects for dependence between means. Cohen's *d* is interpreted as small (0.2), moderate (0.5), and large (0.8) effect size (Cohen, 1988). For main effects and differences between groups, partial eta squared was used as measure of effect size. Partial eta squared is interpreted as small (0.01), medium (0.06), and large (0.14) effect size (Richardson, 2011).

It was of interest to examine differences between responders and non-responders to intervention in order to find out for whom the intervention worked and why. Response to intervention was defined as at least 35.0% improvement in primary outcome symptom measures (PHQ-9 and GAD-7) pre- to post-intervention. ANOVAs were run to compare non-responders and responders in the ATT- and MSC-group on change scores in mindfulness, self-compassion, and attention flexibility. For an easier to interpret graphic presentation, individual change scores on all theoretical measures were rescaled to a standardized 0–100 scale.

Finally, linear regression analyses were conducted using the total sample to predict primary outcome measures post-intervention, using primary outcome measures pre-intervention, age, gender, and change in mindfulness, self-compassion, and attention flexibility as predictors. This was done in order to determine which of these variables contributed to treatment response across conditions.

## Results

### Sample characteristics

An overview of demographics, symptoms, and other measures in the two experimental groups pre-intervention is presented in Table 1. One-way ANOVA indicated no significant differences between groups on any measures pre-intervention. None of the participants reported no symptoms (scores of 0 on all measures). Categorizing symptoms into none, mild, and moderate to severe symptoms, 37.0% showed no symptoms of depression, while 63.0% scored in the mild to severe range. As for symptoms of anxiety, 35.8% showed no symptoms, while 64.2% scored in the mild to severe range. A total of 39.0% reported being a little to very lonely, 21.9% reported having a little bad to bad self-esteem, and 50.6% reported having some to a lot test anxiety.

**Table 1:** Descriptive statistics and comparison between groups pre-treatment (*N* = 81).

	**ATT**	**MSC**	**Total**	***F/x*^2^**	**Sig**.
*N*	40	41	81		
Age	22.7 (3.2)	23.0 (3.4)	22.9 (3.3)	0.20	0.660
Female gender	75.0% (30)	75.6% (31)	75.3% (61)	0.00	0.949
Partner	55.0% (22)	56.1% (23)	55.6% (45)	0.01	0.921
PHQ-9	6.7 (4.3)	7.5 (5.2)	7.1 (4.7)	0.56	0.456
GAD-7	6.0 (2.9)	7.1 (4.1)	6.6 (3.6)	1.94	0.167
SCS-SF	34.5 (9.8)	34.3 (7.5)	34.4 (8.6)	0.02	0.899
DMQ flexibility	15.5 (4.8)	14.1 (4.3)	14.8 (4.6)	1.77	0.187
FFMQ	66.2 (10.4)	62.7 (8.2)	64.4 (9.5)	2.81	0.098
Test anxiety	2.6 (0.7)	2.5 (0.7)	2.5 (0.7)	0.06	0.812
Self-esteem	2.9 (0.8)	2.8 (0.7)	2.8 (0.8)	0.02	0.904
Loneliness	2.7 (0.7)	2.6 (0.6)	2.7 (0.7)	0.60	0.443

*Parenthesis indicate standard deviations, except from female gender and partner, where parenthesis indicate exact values. ATT, Attention Training Technique; MSC, Mindful Self-Compassion; PHQ-9, The Patient Health Questionnaire 9 (range: 0–27); GAD-7, Generalized Anxiety Disorder-7 (range: 0–21); SCS-SF, Self-Compassion Scale Short Form (range: 12–60); DMQ flexibility, Attention Flexibility (range: 5–25); FFMQ, The Five Facet Mindfulness Questionnaire (range: 20–100)*.

### Treatment response

Repeated measures ANOVAs indicated a significant reduction in depressive symptoms (PHQ-9) and anxiety symptoms (GAD-7) in both groups pre- to post-intervention, with medium effect sizes (range: *d* = 0.53–0.71) and no significant differences between conditions (see Table 2). These results were stable, with no significant differences in symptom level between post-intervention and follow-up either for PHQ-9 (*p* = 0.872) or GAD-7 (*p* = 0.934). As presented in Table 3, participants reporting no symptoms increased in both groups for PHQ-9 (ATT = 40.0–55.0%; MSC = 34.1–53.7%) and GAD-7 (ATT = 37.5–60.0%; MSC = 34.1–51.2%), pre- to post-intervention. There was also a substantial decrease in participants scoring within the moderate to severe range. In general, these results were maintained at follow-up, as depicted in Table 3. However, there was a slight increase in participants scoring within the mild range. Defining treatment response by minimum 35.0% reduction in symptoms measures, there was a treatment response post-intervention of 33.3% (GAD-7) and 32.1% (PHQ-9) across conditions. This was maintained at follow-up (GAD-7 = 37.0%; PHQ-9 = 35.8%).

**Table 2:** Summary of means and standard deviations pre-intervention, post-intervention, and follow-up.

				**Pre- to post-intervention**				
					**Main effect**		**Between groups**	
**Variable**	**Pre-score Mean (*SD*)**	**Post-score Mean (*SD*)**	**Follow-up Mean (*SD*)**	***d***	***$\eta_{p}^{2}$***	**Sig**.	***$\eta_{p}^{2}$***	**Sig**.
PHQ-9 ATT	6.7 (4.3)	5.2 (3.7)	5.3 (0.7)	0.53	0.233	<0.001^***^	0.001	0.803
PHQ-9 MSC	7.5 (5.2)	5.9 (5.2)	5.7 (0.7)	0.57				
GAD-7 ATT	6.0 (2.9)	4.4 (2.9)	4.4 (0.5)	0.71	0.278	<0.001^***^	0.001	0.732
GAD-7 MSC	7.1 (4.1)	5.7 (4.2)	5.8 (0.5)	0.54				
SCS-SF ATT	34.5 (9.6)	39.2 (8.0)	39.9 (8.8)	0.82	0.313	<0.001^***^	0.009	0.402
SCS-SF MSC	34.3 (7.4)	37.8 (8.1)	38.1 (8.5)	0.55				
DMQ flexibility ATT	15.5 (4.8)	17.4 (3.9)	17.6 (4.2)	0.51	0.264	<0.001^***^	0.001	0.844
DMQ flexibility MSC	14.1 (4.2)	16.2 (4.6)	16.3 (4.5)	0.73				
FFMQ ATT	66.2 (10.3)	70.4 (9.8)	71.9 (10.8)	0.66	0.262	<0.001^***^	0.004	0.571
FFMQ MSC	62.7 (8.1)	66.1 (9.4)	67.7 (9.3)	0.53				
Self-esteem ATT	2.6 (0.8)	3.0 (0.8)	3.1 (0.8)	0.27	0.062	0.025^*^	0.000	0.978
Self-esteem MSC	2.8 (0.7)	3.0 (0.7)	3.0 (0.7)	0.24				
Test anxiety ATT	2.6 (0.8)	2.4 (0.7)	2.3 (0.9)	0.24	0.048	0.050^*^	0.001	0.807
Test anxiety MSC	2.5 (0.7)	2.4 (0.6)	2.2 (0.6)	0.20				
Loneliness ATT	2.7 (0.7)	2.8 (0.7)	3.0 (0.6)	0.12	0.001	0.757	0.010	0.370
Loneliness MSC	2.6 (0.6)	2.6 (0.6)	2.7 (0.5)	0.07				

*Effect sizes and repeated-measures analysis of variances (ANOVA) results showing effect of intervention and differences in effect between groups pre- to post-intervention*.

*** *p < 0.001*,

* *p ≤ 0.05*.

*ATT, Attention Training Technique; MSC, Mindful Self-Compassion; PHQ-9, Patient Health Questionnaire 9; GAD-7, Generalized Anxiety Disorder-7; SCS-SF, Self-Compassion Scale Short Form; DMQ flexibility, Attention Flexibility; FFMQ, Five Facet Mindfulness Questionnaire*.

**Table 3:** Frequencies of participants showing no, mild, and moderate to severe symptoms of depression and anxiety as measured with PHQ-9 and GAD-7 pre-intervention, post-intervention, and follow-up.

	**PHQ-9 (*****n*****/%)**			**GAD-7 (*****n*****/%)**		
**Cut-off**	**Pre**	**Post**	**FU**	**Pre**	**Post**	**FU**
**ATT**						
0–4 (No symptoms)	16/40.0	22/55.0	20/50.0	15/37.5	24/60.0	21/52.5
5–9 (Mild)	12/30.0	12/30.0	15/37.5	18/45.0	13/32.5	17/42.5
>10 (Moderate to severe)	12/30.0	6/15.0	5/12.5	7/17.5	3/7.5	2/5.0
**MSC**						
0–4 (No symptoms)	14/34.1	22/53.7	21/51.2	14/34.1	21/51.2	20/48.8
5–9 (Mild)	16/39.1	13/31.7	14/34.2	17/41.5	14/34.2	16/39.0
>10 (Moderate to severe)	11/26.8	6/14.6	6/14.6	10/24.4	6/14.6	5/12.2

*ATT, Attention Training Technique; MSC, Mindful Self-Compassion; PHQ-9, Patient Health Questionnaire 9; GAD-7, Generalized Anxiety Disorder-7; FU, 6-month follow-up*.

As presented in Table 2, there was a significant increase in self-compassion (SCS-SF), attention flexibility (DMQ flexibility), and mindfulness (FFMQ) in both groups pre- to post-intervention, with large effect sizes and no significant differences between groups. With small to medium effect sizes, there was also a significant increase in self-esteem and a significant decrease in test anxiety, with no significant differences between groups. For self-compassion, attention-flexibility, and self-esteem, these results were maintained with no significant differences between post-intervention and follow-up (SCS-SF: *p* = 0.356; DMQ flexibility: *p* = 0.618; self-esteem: *p* = 0.358). For mindfulness, there was a significant increase (FFMQ: *p* = 0.010) and for test-anxiety there was a significant decrease (*p* = 0.003) from post-intervention to follow-up. The replacement of missing follow-up data did not affect the results of the analysis as opposed to analyses with completers only.

#### Homework

During the intervention period, the ATT-group listened to the audiotape at home 10.6 times (*SD* = 2.8), compared to the MSC-group in which the participants practiced 7.3 times (*SD* = 2.5). Although this difference in homework is statistically significant [*t*_(66)_ = 5.17, *p* < 0.001], split-plot ANOVAs using homework as a covariate indicated that number of exercises had no significant effect on PHQ-9 (*p* = 0.555) or GAD-7 (*p* = 0.935). Note that missing homework-data was not replaced.

#### Comparison of responders and non-responders

**Figure 2:**
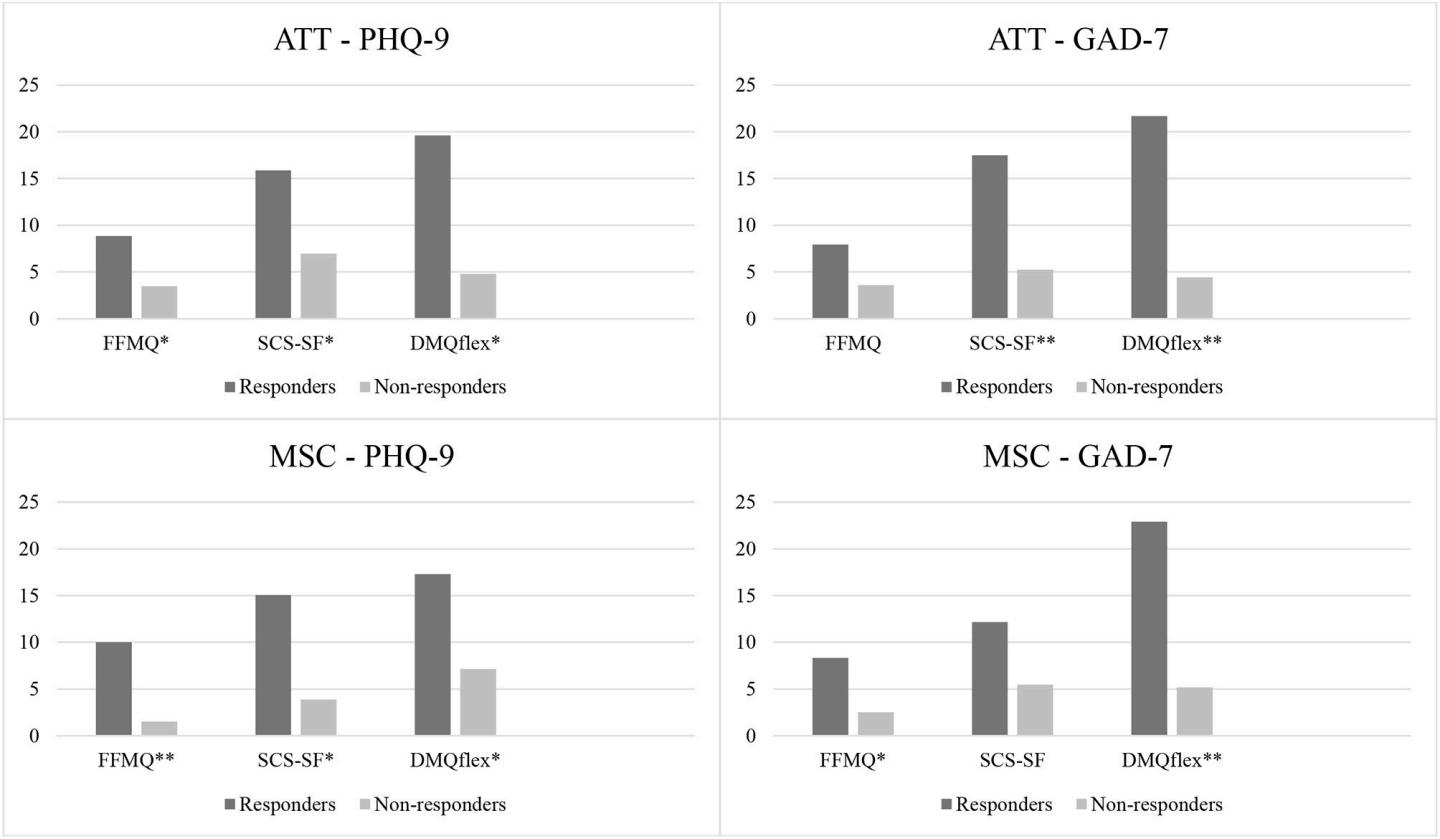
Differences in change scores pre- to post-intervention for responders and non-responders for ATT (*n* = 40) and MSC (*n* = 41) classified by primary outcome measures PHQ-9 and GAD-7. Change scores are reported using transformed scores (0–100). Significant differences between responders and non-responders are highlighted (^*^p < 0.05, ^**^p < 0.01). ATT, Attention Training Technique; MSC, Mindful Self-Compassion; PHQ-9, Patient Health Questionnaire 9; GAD-7, Generalized Anxiety; FFMQ, Five Facet Mindfulness Questionnaire; SCS-SF, Self-Compassion Scale Short Form; DMQflex, Attention Flexibility.

Figure 2 presents a comparison of the change scores (0–100) in mindfulness (FFMQ), self-compassion (SCS-SF), and attention flexibility (DMQflex) for responders and non-responders in the ATT- and MSC-group pre- to post-intervention. ANOVAs indicated significant differences in change scores between responders and non-responders on most of these variables in both the ATT- and the MSC-group, as illustrated in Figure 2. Responders consistently showed higher change scores than non-responders on self-compassion, attention flexibility, and mindfulness.

#### Test of theoretical models

Regression analyses were run in order to determine which variables contributed to explain symptoms post-intervention. Table 4 summarizes results of regression analyses with post-GAD-7 and post-PHQ-9 scores as outcome variables, respectively. For post-PHQ-9 the total model explained a significant proportion of variance, *R*^2^ = 0.696, *F*_(6, 74)_ = 31.58, *p* < 0.001. For post-GAD-7 the model explained a significant proportion of the variance, *R*^2^ = 0.664, *F*_(6, 74)_ = 27.34, *p* < 0.001. Change in attention flexibility (DMQ flexibility) was the only unique theoretical variable that significantly predicted treatment outcome.

**Table 4:** Multiple regression analyses with post-intervention PHQ-9 and GAD-7 regressed on pre-intervention PHQ-9 and GAD-7 respectively, as well as age, female gender, and change in SCS-SF, change in DMQ flexibility, and change in FFMQ across experimental groups.

**Variable**	**ß**	***T***	**Sig**.
**PHQ-9 POST**			
Age	0.009	0.140	0.889
Female gender	−0.056	−0.860	0.393
PHQ-9 pre	0.805	12.824	0.000^**^
SCS-SF Δ	0.000	−0.006	0.996
DMQ flexibility Δ	0.193	2.582	0.012^*^
FFMQ Δ	0.085	1.137	0.259
**GAD-7 POST**			
Age	−0.100	−1.465	0.147
Female gender	−0.088	−1.280	0.205
GAD-7 pre	0.814	11.824	0.000^**^
SCS-SF Δ	−0.014	−0.161	0.872
DMQ flexibility Δ	0.214	3.091	0.003^**^
FFMQ Δ	0.050	0.639	0.525

** *p < 0.01*,

* *p < 0.05*.

*PHQ-9, Patient Health Questionnaire 9; GAD-7, Generalized Anxiety Disorder-7; SCS-SF, Self-Compassion Scale Short Form; DMQ flexibility, Attention Flexibility; FFMQ, Five Facet Mindfulness Questionnaire*.

## Discussion

The aim of the present study was to examine the effectiveness of a 3-week intervention trial based on either MSC or ATT for symptoms of anxiety and depression among students, and to investigate two theoretical models for emotional disorder. Both interventions were associated with reduction in symptoms of anxiety and depression, as predicted by the first hypothesis. In support of the second hypothesis, both interventions significantly increased mindfulness, attention flexibility, and self-compassion pre- to post-intervention. The changes predicted by both hypotheses were maintained when measured at 6-month follow-up. A clear trend was found when comparing responders and non-responders to intervention: responders showed larger change-scores than non-responders on all theoretical measures pre- to post-intervention. This might indicate that all of these are possible change mechanisms. However, change in attention flexibility was the only unique predictor of treatment outcome.

In comparison with relevant previous studies (Fergus et al., 2014; Nassif and Wells, 2014; Smeets et al., 2014; Callinan et al., 2015; McEvoy et al., 2017), all effect sizes in the current study were similar or even larger for primary outcome measures. This indicates that the interventions were administered in a satisfactory manner, and that both ATT and MSC performed in groups were equally effective in reducing depressive and anxiety symptoms among non-clinical individuals. Furthermore, stability in this reduction 6 month later indicates that such interventions may buffer against development of emotional disorder. These results support the potential of ATT and MSC as standalone interventions and imply that they are suitable for group-administration. As for the optimal practice dosage, six to nine sessions of ATT have previously been proposed to attain long-term effects (Knowles et al., 2016). The current study, however, found that three sessions might be sufficient and that the amount of home practice between sessions was not important for symptom reduction in either the ATT- or MSC-group. Hence, understanding the rationale is probably more important than the amount of practice, and reaching this comprehension might be the main function of practicing. However, this assumption needs further investigation. Altogether, the support of the first hypothesis suggests that MSC and ATT performed as 3-week group interventions might produce beneficial and lasting effects for non-clinical student samples.

In support of the second hypothesis, self-compassion significantly increased in both groups. This indicates that both interventions may strengthen the capability to relate to oneself in a friendlier manner. The increase in self-compassion was expected in the MSC-condition based on previous studies (Neff and Germer, 2013; Smeets et al., 2014) and as this intervention had an explicit focus on self-compassion. Self-compassion has also been suggested a central change mechanism following mindfulness interventions (Shapiro et al., 2005; Germer and Neff, 2013). As for the ATT-group, the increase in self-compassion is particularly interesting, as this is not an explicit aim of this technique. A possibility is that ATT may strengthen the ability to voluntarily change the attentional focus away from self-criticism.

Mindfulness also increased significantly pre- to post-intervention in both groups and was the only theoretical measure that significantly increased from post-intervention to follow-up. Even though increase in mindfulness is not originally predicted following ATT, Wells (2002) acknowledges that there are similarities between mindfulness and ATT. This is plausible as both techniques support distancing from mental and external events, allowing thoughts and feelings to come and go, and promote present-moment focus through formal exercises. The current study supports this notion and suggests that although deriving from different conceptual frameworks, both interventions affect related skills that keep developing months after intervention. Increase in mindfulness in both groups also supports previous statements that mindfulness and metacognitions may share overlapping elements (Solem et al., 2015).

Of all the theoretical measures included, change in attention flexibility was the most unique predictor of symptom reduction post-intervention. Enhanced attention flexibility was expected in the ATT-group as this is an explicit goal of the technique, and the results support previous studies (Nassif and Wells, 2014; Callinan et al., 2015). This also yields support for the metacognitive model of emotional disorder in that attentional control is important in disrupting the CAS. The current results also expand previous claims of the relative contribution of attention in mindfulness-based interventions (e.g., Jha et al., 2007). Mindfulness-based exercises might enhance attention flexibility by training the ability to shift attentional focus between the breath and other sensations, while inhibiting distractions. Altogether, the results suggest that both ATT and MSC strengthen the capacity to respond to one's internal and external environment in a more flexible manner.

This may at first glance seem inconsistent with the findings of McEvoy et al. (2017), who found that reduction in anxiety was independent of improvement in cognitive flexibility as measured by emotional Stroop. However, this inconsistency is probably due to methodological challenges already outlined by McEvoy et al. (2017), that the emotional Stroop-task may have been unable to detect changes in attention flexibility following intervention. Therefore, the flexibility subscale of DMQ may be more suitable than emotional Stroop in examining the contribution of attention flexibility in symptom reduction.

One implication from the current study is that techniques developed within different theoretical frameworks may decrease symptoms of anxiety and depression via common mechanisms. All theoretical measures increased similarly across both conditions and scores were higher among responders than non-responders to interventions. Several studies comparing different psychotherapies has found equal symptom reduction and improvements on both model-specific as well as common factors (e.g., Warmerdam et al., 2010; Lemmens et al., 2017), indicating that psychological processes necessary for symptom reduction seems comparable across theoretical background. Although the current study does not imply causality, it suggests attention flexibility might be an efficacious underlying mechanism of change across interventions and a potent common factor between ATT and MSC.

It is further interesting to discuss the relationship between direction of attentional focus and attention flexibility, as we would argue that these constructs seem somewhat overlapping. Fergus et al. (2014) found that the function of self-focused attention may vary depending on the context in which it is performed, thus supporting the differentiation between subtypes of self-focused attention such as ruminative or self-critical, vs. experiential or mindful self-focus (Baer, 2009). This notion was not fully supported in a later study, where no relationship between locus of attention and symptom reduction was found (McEvoy et al., 2017). The authors concluded that locus of attention may be less important than the ability to distance oneself from one's experiences, the capacity of present-moment attention, and an experience of control over one's attention (McEvoy et al., 2017). Based on the unique role of attention flexibility in the current study, it seems plausible that flexibility in attention and voluntary control may be more important than whether the focus is internal or external. This relationship should be empirically investigated.

Limitations in this study should be considered. Participants were not assessed with diagnostic interviews and therefore diagnostic precision is lacking. There is also a possibility of selection bias, as all participants actively signed up for the study and might have a particular interest in the methods or topics. The majority of the participants were also female psychology students. Further, previous research complicated power calculations due to incomparable measure instruments. The between group comparisons suggested no clear trend that either intervention should be superior to the other. Given these results, a sample size of 400+ would be needed for a significant difference to be detected between the two interventions. Although the sample size was not large, it was still designed to be appropriate for detecting small differences (Whitehead et al., 2016).

Also, all outcome measures were based on self-report and short forms were used for two out of five measures (FFMQ and SCS-SF). Concerning therapist competence, a limitation is that the group leaders had not previously been practicing mindfulness or ATT on a regular basis. Further, the sessions were not videotaped and thus cannot be evaluated. However, administration was mainly automatized using pre-recorded audiotapes and the results indicate that both conditions were associated with significant symptom reduction. The lack of long personal experience with either intervention may also have been beneficial in that the therapists had no prior preference for ATT or MSC and as such were unbiased. Due to the study design, it remains unknown whether symptom reduction is caused mainly by the specific intervention techniques or other therapeutic or extra-therapeutic factors. Possible mechanisms include common factors, pleasing, sharing experiences in a group, and expectancy effects. These were not controlled for in the current study. An improvement of the study design would therefore include a third condition consisting of an attention placebo. Without such a condition it is difficult to conclude as to the specific treatment effects of the two conditions.

A possible limitation is that MSC could be biased toward being attentive to suffering. Therefore, results could have been different using other types of mindfulness interventions such as mindful meditation. Several studies have supported use of mindful meditation for improving attentional control, while few studies have investigated this for MSC. However, as the results showed, both MSC and ATT were equally efficient in improving attention flexibility thereby demonstrating that also MSC is associated with change in attention. It has been suggested that self-compassion could be a better predictor of symptom severity than mindfulness (Van Dam et al., 2011). However, other studies have not reached the same conclusion (e.g., López et al., 2016). Further research is therefore needed in order to discover whether any specific type of intervention is more efficient than others in improving attention flexibility.”

A practical implication from the current study is that attention flexibility might be an important common factor in emotional disorders and treatment. The importance of these findings is mirrored by observations that techniques enhancing skills of attentional control (e.g., by mindfulness meditation) could prevent relapse in depression (Teasdale et al., 1995). This finding has considerable implications and provides an initial step toward understanding how attention flexibility could be a possible mechanism by which interventions may decrease symptoms of depression and anxiety. Future studies should investigate the most efficient ways of improving attentional control. It is therefore important for future research to examine *how* and *why* attention flexibility works, in order to optimize treatment by specifically targeting this underlying mechanism. Additional and more objective measures of attention flexibility than self-report should be included, such as set-shifting tasks. The relationship between attention flexibility and locus of attention is also of interest to investigate. Future studies may also include an additional control group such a wait-list or a talking condition.

## Conclusion

This RCT supports both MSC and ATT as promising perspectives for reducing symptoms of anxiety and depression when administered in a brief group based intervention. Symptom reduction was accompanied by significant increases in mindfulness, self-compassion, and attention flexibility post-intervention. These results were maintained at 6-month follow-up and the level of mindfulness even kept increasing from post-intervention to follow-up. Mechanisms of change may be more similar across the techniques than different, and increase in attention flexibility may be the most important underlying psychological process in both models. Thus, targeting attention flexibility specifically should be of interest in psychological treatment and future research.

## Ethics statement

The study was a RCT approved by the Regional Medical Ethics Committee in Norway (ref.nr. 2015/470). Informed written consent was given from all participants.

## Author contributions

SS initiated the project and was responsible for getting ethical approval. HH and GV were group leaders for the first four groups. IG and RH were group leaders for the last six groups. SS supervised the group leaders. All authors contributed equally to writing the manuscript and analyzing the data.

### Conflict of interest statement

The authors declare that the research was conducted in the absence of any commercial or financial relationships that could be construed as a potential conflict of interest.

## Footnotes

^1^As effect sizes were not given by Fergus et al. (2014), these were calculated using Morris and DeShon's (2002) Equation (8) with correlation coefficient 0.5.
